# Weight-Bearing Approaches After Neck of Femur Fractures: A Narrative Review of Evidence and Outcomes

**DOI:** 10.7759/cureus.84932

**Published:** 2025-05-27

**Authors:** Niketa Patel, Mahima Chaudhari

**Affiliations:** 1 Department of Physiotherapy, College of Physiotherapy, Sumandeep Vidyapeeth Deemed to be University, Vadodara, IND

**Keywords:** fracture neck of femur, hip fracture, mobilization, post-operative rehabilitation, weight bearing

## Abstract

Neck of femur fractures, a prevalent injury among the elderly, significantly impair mobility, independence, and quality of life. The timing and extent of weight bearing post-surgery are critical to recovery, yet clinical practices vary widely. This narrative review synthesizes evidence on weight-bearing strategies following neck of femur fractures, focusing on their impact on functional outcomes, complications, and hospital stay duration. A comprehensive literature search was conducted using PubMed, Science Direct, and Google Scholar for studies published between 2012 and 2024. The keywords, along with the Boolean operators utilized, consisted of “Neck of femur fracture”, OR “Hip fracture”, AND “Weight bearing”, AND “Mobilization”, OR “Mobility”, OR “Ambulation”, OR “Gait training”, to inculcate appropriate literature. The studies reported that early weight bearing (within 24 to 48 hours post-surgery) and full weight bearing are strongly supported for enhancing mobility, reducing hospital stays, and mitigating complications like pneumonia, pressure ulcers, and deep vein thrombosis. Partial weight bearing, while practiced, is less effective due to poor compliance in geriatric patients, often leading to immobility. Delayed and non-weight-bearing approaches are associated with prolonged recovery and increased complications. Fracture type, surgical approach, and patient characteristics influence optimal strategies. In conclusion, early full weight bearing is the preferred approach for geriatric patients’ post-neck of femur fractures, promoting functional recovery and reducing complications. However, standardized protocols are needed to address practice variability.

## Introduction and background

Advancements in healthcare and biotechnology have significantly reduced mortality rates, leading to increased life expectancy worldwide [1]. However, this demographic shift has brought challenges, notably a rise in age-related conditions such as hip fractures, which disproportionately affect the elderly [2]. Hip fractures, particularly neck of femur fractures, are a major public health concern due to their impact on mobility, independence, and quality of life [1,2]. These fractures often result from low-energy trauma in older adults, who are already prone to reduced mobility and dependency in activities of daily living (ADLs) [2]. The consequences are profound, as approximately one-third of individuals who sustain a hip fracture do not survive beyond one year, highlighting the severity of this injury [3].

Neck of femur fractures, a subtype of hip fractures, are the most common traumatic injuries in the elderly, driven by age-related bone loss in the femoral neck, an intracapsular region of the femur [4]. These fractures are classified as displaced or undisplaced, with treatment strategies varying accordingly. Undisplaced fractures may be managed conservatively, while displaced fractures typically require surgical intervention, such as internal fixation or arthroplasty [1]. The choice of treatment is influenced by multiple factors, including the patient’s age, bone quality, and the time elapsed between injury and surgery [5]. Post-surgical recovery is complex, depending on fracture type, surgical technique, implant fixation, bone health, muscle strength, and pre-fracture functional status [6].

Rehabilitation is a cornerstone of recovery following neck of femur fractures, encompassing not only physiotherapy but also occupational therapy, psychological support to restore confidence, and nutritional therapy to optimize healing [7]. Physiotherapy focuses on strength training, mobilization, and gait training to address deficits caused by the fracture and surgery [4,8]. A critical aspect of rehabilitation is the timing and extent of weight bearing, which significantly influences functional outcomes. Prolonged immobilization and bed rest have well-documented adverse effects, including bone loss, muscle atrophy, and systemic complications such as pressure ulcers, urinary tract infections, pneumonia, and deep vein thrombosis (DVT) [2,9]. Early mobilization can mitigate these risks, promoting faster recovery and reducing the likelihood of falls and further immobilization [9,10].

Historically, concerns about implant or fixation failure led surgeons to recommend partial weight bearing or delayed weight bearing to protect osteosynthetic fixation [10]. Despite evidence that early weight bearing reduces morbidity and mortality risks in orthopaedic surgeries [11], approximately 25% of surgeons continue to advocate for partial or delayed weight bearing, reflecting a lack of global consensus [9]. This variability in practice is evident worldwide, with significant implications for patient outcomes [12]. Patients who engage in early weight bearing typically achieve independence more quickly, resulting in shorter hospital stays, which is a key indicator of successful recovery [1,4].

While studies on total hip arthroplasty have demonstrated that early weight bearing does not adversely affect implant stability [13-15], research specifically addressing early weight bearing after neck of femur fractures is limited, and opinions diverge on the optimal duration for restricting weight bearing [16]. This gap in evidence underscores the need for a comprehensive synthesis of current knowledge to guide clinical practice and improve patient outcomes. This narrative review aims to provide a detailed overview of the current state of knowledge on weight-bearing strategies following neck of femur fractures, with a focus on their impact on functional recovery, complications, and hospital stay duration.

## Review

**Figure 1:**
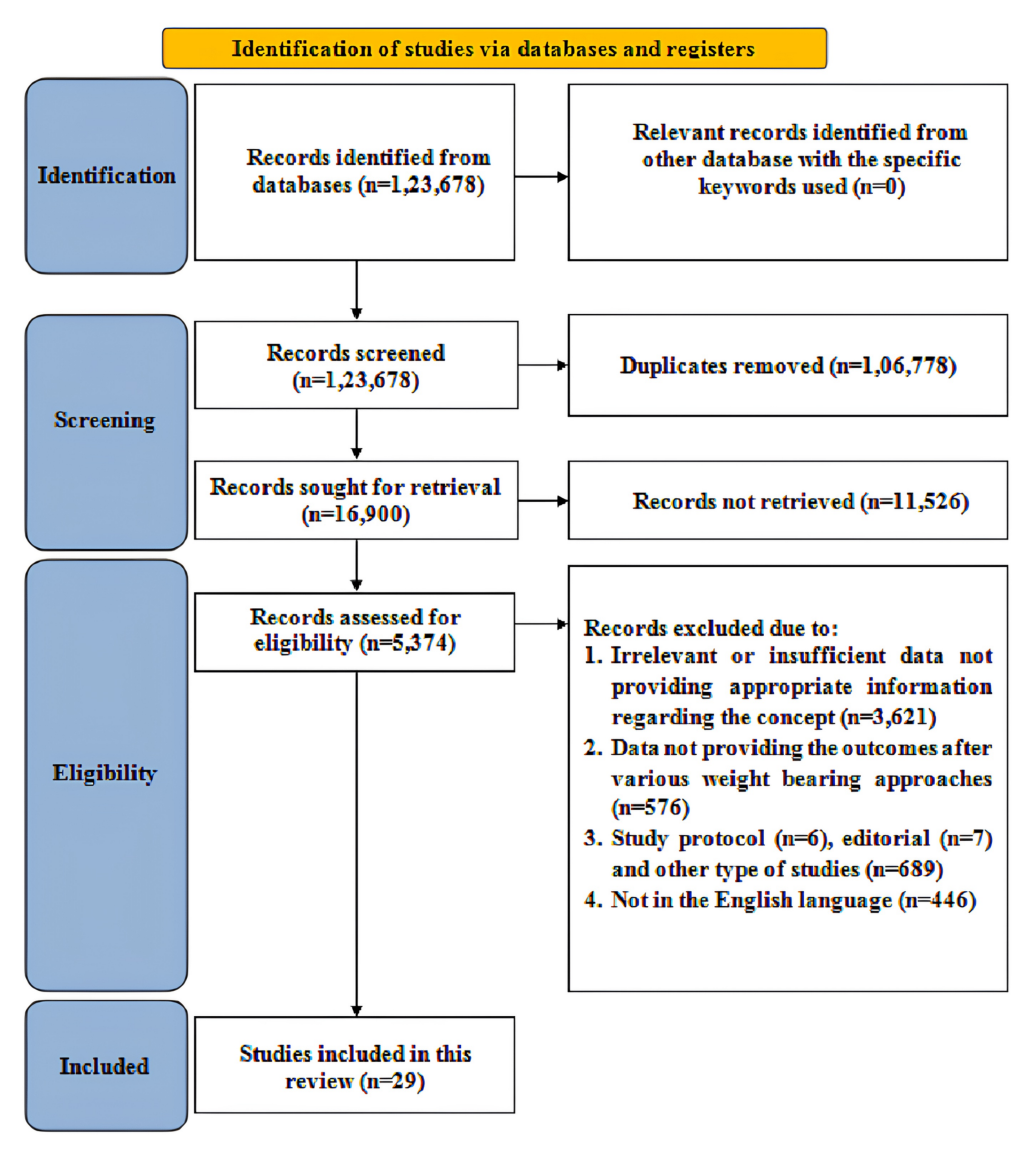
PRISMA flowchart describing search strategy PRISMA = Preferred Reporting Items for Systematic Reviews and Meta-Analysis

The Scale for the Assessment of Narrative Review Articles (SANRA) was utilized to search the articles, formulate inclusion and exclusion criteria, critically appraise the literature, and appropriately present the evidence [17]. Additionally, the Preferred Reporting Items for Systematic Reviews and Meta-analyses (PRISMA) framework, as illustrated in Figure 1, was utilized for the identification, screening, selection, and synthesis of studies [18].

Data sources and search strategy

A comprehensive literature search was performed on Science Direct, PubMed, and Google Scholar databases with keywords, along with the Boolean operators that consisted of “Neck of femur fracture”, OR “Hip fracture”, AND “Weight bearing”, AND “Mobilization”, OR “Mobility”, OR “Ambulation”, OR “Gait training”, to inculcate appropriate literature from 2012 to 2024 to ensure relevance to contemporary clinical practices and recent advancements in orthopaedic and rehabilitation strategies.

Study screening and selection

The inclusion criteria for screening were studies in English with full-text availability that focused on weight bearing (early, full, partial, delayed, or non-weight bearing) after neck of femur fractures, though relevant hip fracture studies were also included. Additionally, studies examining weight bearing as a primary or secondary outcome, including its impact on mobility, functional recovery, complications, or hospital stay duration, studies involving adult patients, with a preference for geriatric populations (typically aged 60 years or older), as neck of femur fractures are most prevalent in this group, studies including patients who underwent surgical intervention (e.g., internal fixation, hemiarthroplasty, or total hip arthroplasty) for neck of femur or hip fractures, and study designs involving prospective and retrospective cohort studies, randomized controlled trials (RCTs), meta-analyses, review studies, case reports, and experimental studies were also included. Moreover, studies reporting outcomes related to weight bearing, such as functional recovery (e.g., mobility, gait, activities of daily living), complications (e.g., pneumonia, pressure ulcers, deep vein thrombosis), hospital stay duration, mortality rates, and implant stability or surgical outcomes, and studies that discussed physiotherapy, mobilization, or rehabilitation protocols in the context of weight bearing post-fracture, as these are integral to recovery were included.

However, articles that did not address weight bearing or its outcomes after neck of femur or hip fractures (e.g., studies focusing solely on surgical techniques, fracture classification, or unrelated rehabilitation aspects), and studies primarily addressing non-hip fractures (e.g., distal femur, tibial, or upper limb fractures) unless they provided relevant insights into weight bearing principles applicable to hip fractures were excluded. Additionally, articles with only a title and no abstract, studies not published in English or lacking an available English translation, those with unavailable full text, and those providing insufficient contextual information were excluded. 

Two independent reviewers evaluated the articles to determine their suitability for inclusion in the review. Initially, titles and abstracts were screened to remove duplicates. Next, the selected articles were re-screened to exclude those that did not meet the eligibility criteria. Finally, the remaining articles were assessed based on their full text to confirm eligibility. Any discrepancies or disagreements between the reviewers were resolved through discussion and consensus. The critical narrative approach was used to synthesize the results of the included studies [19]. The studies utilized various research methodologies and outcome measures, resulting in a considerable level of heterogeneity.

Data extraction and synthesis

Data was extracted based on the information consisting of evidence reporting effectiveness and restrictions of weight-bearing rehabilitation outcomes post neck of femur fractures. The critical narrative technique was employed to integrate text and figures to summarize and validate evidence.

Effectiveness and restrictions of various weight-bearing approaches

Full Weight Bearing

Full weight bearing, often initiated early, is associated with superior functional outcomes and practical advantages for elderly patients. Kim et al. (2014) found that geriatric patients with neck of femur fractures who engaged in early full weight bearing regained pre-injury ambulation levels and had lower mortality rates [5]. Another study supporting the full weight bearing was done on operated hip fractures in the geriatric population. The study suggested that adhering to and understanding partial weight bearing is difficult for the geriatric population, for which it would be easy for the geriatric population to adhere to full weight bearing, and it would improve the functional recovery [15]. Similarly, Pfeufer et al. (2019) conducted a pre-post study comparing partial (n=19) and full (n=22) weight-bearing groups, finding that restricted weight bearing reduced gait speed and mobility, as patients often failed to comply with restrictions, leading to complications. The study, which used insole force sensors starting on post-operative day (POD) 5, recommended early full weight bearing even in geriatrics [9].

Additionally, a meta-analysis by Tian et al. (2017), which analysed results from nine studies (RCTs and non-RCTs) on uncemented total hip arthroplasty (THA) further supported full weight bearing, reporting improved Harris Hip Scores and no increased risk of implant loosening [14]. While this study did not directly examine neck of femur fractures, its findings contribute indirect support to the safety and benefits of early full weight bearing. Hence, these collective findings suggest that full weight bearing is not only safe but also optimizes recovery, particularly when initiated early, aligning with the goal of achieving ambulatory independence.

Partial Weight Bearing

Partial weight bearing remains a common practice in some settings to protect surgical fixation, but its efficacy and feasibility are questioned, especially in geriatric populations. Baer et al. reviewed hemiarthroplasty for displaced neck of femur fractures, suggesting that partial weight bearing is preferable to non-weight bearing as it promotes ambulation and reduces immobilization-related complications [15]. Rehabilitation starts with bed exercises during the immobilization phase after surgery and progresses to partial weight bearing for eight weeks. Similarly, Kubiak et al. (2013) noted that partial weight bearing transfers loads to the fracture site, aiding bone strength. At least in partial weight bearing, the patient will ambulate and thus avoid bedridden (immobilization) complications. Also, the operated fracture site will receive a certain amount of loads, and forces will be transferred from this, which will help in improving strength at the fracture site [20].

However, Kammerlander et al. (2018) used insole force sensors to compare weight bearing in geriatric (≥75 years) and younger (18-40 years) patients, finding that elderly patients were unable to comply with prescribed partial weight bearing, leading to immobility and complications. The authors advised against postoperative partial weight bearing in geriatrics [21]. Hurkmans et al. (2012) conducted an RCT evaluating audio feedback for partial weight bearing training in THA patients, finding it effective in younger adults but less applicable to geriatrics due to compliance issues [22]. Moreover, another study conducted, comparing partial and full weight bearing, involved a meta-analysis that included six RCTs and three non-RCTs. The results demonstrated that the Harris Hip Score had improved in full weight-bearing patients. Hence, the author concluded that full weight bearing is very important in total hip replacement (THR), that it does not lead to complications like loosening of the implants, and that it provides better functional outcomes [14]. Furthermore, Xu et al. (2017) described a rehabilitation protocol starting with bed exercises and progressing to partial weight bearing for eight weeks, but this approach may delay recovery compared to early full weight bearing [23]. Hence, these findings suggest that partial weight bearing, while still practiced, is less effective and harder to implement in elderly patients, often leading to adverse outcomes.

Non-weight Bearing

Non-weight bearing is consistently associated with poorer outcomes. Warren et al. (2019) prospectively evaluated early weight bearing as tolerated on POD 1, reporting reduced complications, mortality, and hospital stays compared to non-weight bearing [11], whereas Siebens et al. (2012) supported weight bearing as tolerated in hip replacement patients’ post-fracture, noting improved functionality and shorter hospital stays [24]. A study targeted patients who had undergone an operation for an intertrochanteric femur fracture, a subtrochanteric femur fracture, or a neck femur fracture and assessed the cognitive status, pre-fracture level, ambulatory status, and the need for walking aids in these patients. They followed the patients for one year and concluded that patients’ cognitive status and pre-fracture level played a deep role in achieving functional independence. Additionally, patients’ ambulatory status was increased by the end of the year [25]. However, there are clinical scenarios where non-weight bearing is necessary to protect surgical outcomes or prevent further injury. For instance, in patients with severe osteoporosis, the structural integrity of bone may not support early loading, increasing the risk of implant subsidence or cut-out, particularly after fixation with dynamic hip screws or intramedullary nails [26]. Moreover, a case report by Purushe et al. (2021) described a 60-year-old female post-THA who followed non-weight bearing without adverse effects, suggesting context-specific applicability [27]. Additionally, in unstable femoral neck fractures or periprosthetic fractures, surgeons may prescribe non-weight bearing to avoid displacement or failure of internal fixation [28]. Furthermore, in patients with pathological fractures, such as those due to metastatic disease, restricted or non-weight bearing may be essential due to compromised bone strength [29]. Overall, non-weight bearing is detrimental to functional recovery and should be avoided unless clinically necessary. 

Early Weight Bearing

Early weight bearing, typically initiated within 24 to 48 hours post-surgery, is strongly supported by multiple studies for its benefits in geriatric patients. A prospective cohort study comparing gait in patients with neck of femur fractures versus per-trochanteric fractures was conducted, in which early weight bearing enhanced mobility and was influenced by factors such as type of fracture and the approach of surgery [2]. Similarly, Kuru and Olcar (2020) performed a retrospective analysis consisting of geriatric patients with neck of femur or intertrochanteric fractures, and reported that mobilization within 24 hours improved walking ability; shortened hospital stays, and reduced complications in comparison to partial or delayed weight bearing [10]. These findings align with Kim et al. (2014), who found that early full weight bearing on POD 1 in undisplaced neck of femur fractures led to excellent recovery, restored pre-fracture ambulation, and reduced mortality rates [5].

Moreover, a small sample size pre-post design study was conducted on 19 patients in a group who were prescribed partial weight bearing post surgery, whereas 22 patients in another group were prescribed fully weight bearing as tolerated to analyse whether partial weight bearing had any effect on mobility reduction. The weight bearing started on POD 5 (maximum protection phase), and the outcome consisting of gait was analysed using an insole force sensor. The results reported that post-operative geriatric patients did not follow the early weight-bearing, which resulted in immobility and led to complications [9]. Hence, the finding supported the fact that it is highly recommended to start the early full weight bearing in the post-operative hip fractures, though the population might be geriatric.

Additional studies reinforce these benefits as Shanb and Youssef (2014) emphasized early weight bearing in patients over 70 years to prevent respiratory complications and immobility, advocating for prompt physiotherapy [30]. Similarly, Purushe et al. (2021) supported early walking to mitigate respiratory issues in proximal femur fracture cases [27]. Additionally, Rozell et al. (2016) and Riemen and Hutchison (2016) [22] reviewed hip fracture management, concluding that early weight bearing improves surgical outcomes and reduces hospital stays [31,32]. Moreover, Tarrant et al. (2022) prospectively linked early weight bearing to lower mortality rates, noting that restricted weight bearing should be initiated if full weight bearing is not feasible [33]. Congruently, Perracini et al. (2018) advocated for early mobilization during the maximum protection phase, emphasizing the importance of completing physiotherapy protocols [34].

Furthermore, in a retrospective study by Ariza-Vega et al., it was suggested that immediate weight bearing be allowed for the patients who underwent hip fracture surgery, and patients should be encouraged to perform more weight-bearing activities to avoid complications [12]. Additionally, a meta-analysis was conducted to compare early full weight bearing and partial weight bearing in patients who underwent uncemented THR, for which a total of nine RCTs and non-RCTs focusing on early versus partial weight bearing in uncemented THR surgery were shortlisted. The findings from the study suggested that the Harris Hip Score improved in the patients who were allowed for early full weight bearing, and this did not increase the incidence of complications in the surgical outcomes [14]. Similarly, in a consecutive cohort study, on implementation of non-weight bearing resulted decrease in functional outcome at four months in operated neck femur fractures. This deteriorated status remained the same even at the one-year follow-up [25]. Hence, these studies collectively demonstrate that early weight bearing enhances functional recovery, reduces hospital stays, and mitigates complications, particularly in geriatric populations.

Delayed Weight Bearing

**Figure 2:**
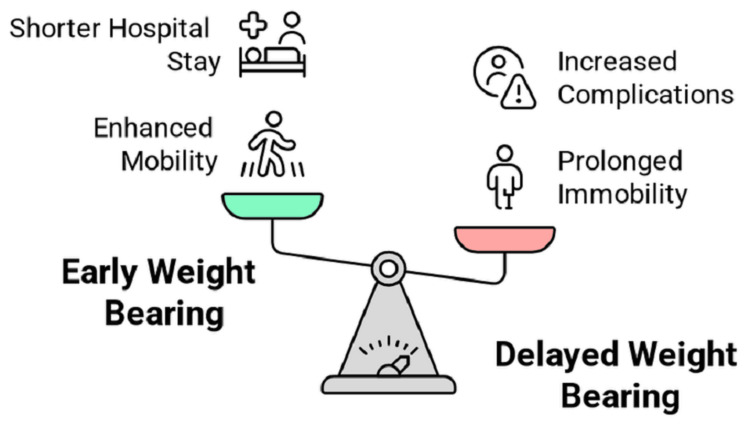
Comparison of outcomes regarding early and delayed weight bearing Image created by the authors.

Evidence on delayed weight bearing (beyond 24 to 48 hours) is sparse, reflecting a shift toward early mobilization. Kubiak et al. (2013) noted a lack of studies supporting delayed weight bearing, suggesting it contributes to prolonged immobility and, therefore, evidence describing its benefits is limited [20]. Kuru and Olcar (2020) retrospectively compared early and delayed weight bearing in geriatric patients with neck of femur or intertrochanteric fractures, finding that delayed weight bearing (after 24 hours) resulted in lower Harris Hip Scores and longer hospital stays. These findings indicate that delayed weight bearing is suboptimal, increasing the risk of complications and delaying recovery [10]. The comparative analysis of outcomes regarding early and delayed weight bearing is illustrated in Figure 2.

Complications of immobilization

Prolonged immobilization, whether due to delayed or non-weight bearing, is linked to serious complications, particularly in geriatric patients. Madhukar and Kumar (2016) retrospectively analyzed hip fracture patients, finding that longer immobilization increased pneumonia risk, a leading cause of respiratory failure and mortality [35]. Similarly, Lv et al. (2015) confirmed that postoperative pneumonia negatively impacts surgical outcomes, emphasizing the need for early mobilization to reduce chest complications [36]. Additionally, Galivanche et al. (2019) and Chiari et al. (2017) conducted prospective cohort studies, identifying prolonged immobilization as a primary cause of pressure ulcers, particularly at the heel. They recommended early mobilization and repositioning every two hours [37,38]. Correspondingly, Dubljanin-Raspopović et al. (2012) linked prolonged bed rest to increased mortality due to pressure ulcers [39]. Whereas, a review study stated that early mobilization is important to avoid pressure sores [40]. Moreover, a prognostic study was carried out on the prevalence of developing pressure sores during the hospital stay [38]. The study findings stated that geriatric patients should be immediately mobilized once the urinary catheter is removed [38]. Furthermore, Kubiak et al. (2020) reviewed post-discharge complications, identifying DVT as a serious consequence of prolonged immobilization [19]. However, early weight bearing is critical to mitigate this risk, as immobility promotes venous stasis.

Synthesis and implications

The evidence strongly supports early full weight bearing as the optimal strategy for neck of femur fracture patients, particularly in geriatric patients. It enhances functional recovery, reduces hospital stays, and mitigates complications like pneumonia, pressure ulcers, and DVT. Full weight bearing is more practical for elderly patients, who often struggle with partial weight bearing compliance, and does not increase implant failure risks. It is important to note that the generalizability of early full weight bearing as a universal approach may be limited by variations in fracture type and surgical method, and clinical decisions must be individualized based on the specific surgical and anatomical context. Partial weight bearing, while still practiced, may delay recovery and increase complications due to non-compliance. Delayed and non-weight-bearing approaches are associated with prolonged immobility, reduced functional independence, and higher complication rates. However, the optimal weight-bearing protocol depends on factors such as fracture type, surgical approach, and patient characteristics (e.g., cognitive status, pre-fracture mobility). Undisplaced fractures may tolerate early full weight bearing better than displaced fractures requiring complex fixation. The lack of standardized guidelines contributes to practice variability, with some surgeons favoring partial weight bearing to protect osteosynthetic fixation despite evidence to the contrary.

Strengths and limitations

This narrative review offers a robust overview of weight-bearing strategies following neck of femur fractures. The inclusion of various study designs allowed for a multifaceted exploration of weight-bearing practices. This diversity captures empirical data and expert perspectives, providing a holistic understanding of early, full, partial, delayed, and non-weight-bearing approaches, as well as associated complications. Moreover, by prioritizing studies involving elderly patients, the review aligns with the epidemiology of neck of femur fractures, which predominantly affect individuals aged 60 and older. This focus ensures clinical relevance, as geriatric patients face unique challenges, such as reduced mobility, cognitive impairments, and higher comorbidity burdens, which influence weight-bearing outcomes. Additionally, a comprehensive outcome analysis approach highlights the multifaceted benefits of early weight bearing and the risks of prolonged immobilization, informing clinical decision-making.

However, the review demonstrates certain limitations that should be considered when interpreting the findings. While the review prioritized studies on neck of femur fractures, some included studies addressed hip fractures more broadly (e.g., intertrochanteric or subtrochanteric fractures) or related procedures like THA. This heterogeneity may limit the specificity of conclusions for neck of femur fractures, as different fracture types and surgical interventions have varying biomechanical and recovery profiles. Additionally, only a few studies were RCTs, with many being observational (cohort studies, case reports) or reviews. Similarly, several studies, particularly cohort studies and case reports, had small sample sizes, which may limit statistical power and generalizability. Small samples are particularly problematic in geriatric populations, where inter-individual variability (e.g., cognitive status, pre-fracture mobility) can significantly influence outcomes. Furthermore, the reviewed studies lacked standardized definitions and protocols for weight bearing (e.g., timing, extent, and duration). For instance, "early weight bearing" ranged from POD 1 to POD 5 across studies, and the distinction between full and partial weight bearing was not always clearly delineated. Lastly, by limiting the review to English-language studies, potentially relevant research published in other languages was excluded, which may have introduced selection bias and overlooked valuable perspectives from non-English-speaking regions.

## Conclusions

This narrative review underscores that early full weight bearing, initiated within 24 to 48 hours post-surgery, may be the optimal strategy for geriatric patients with neck of femur fractures. Existing evidence demonstrates that early weight bearing enhances functional recovery, restores pre-fracture ambulation, reduces hospital stays, and mitigates complications such as pneumonia, pressure ulcers, and deep vein thrombosis. Full weight bearing is particularly effective and practical for elderly patients, who often struggle with partial weight bearing compliance, and does not increase implant failure risks. However, its application should not be generalized across all types of femoral neck fractures or surgical approaches. In contrast, partial weight bearing, though still practiced, delays recovery and increases complications due to non-compliance, while delayed and non-weight bearing approaches are associated with prolonged immobility and poorer outcomes. However, these conclusions are primarily drawn from observational studies, small cohort studies, and case reports, with limited high-quality randomized evidence. Therefore, the optimal weight-bearing protocol depends on factors such as fracture type, surgical approach, and patient characteristics (comorbidities and bone quality), including cognitive status and pre-fracture mobility. The lack of standardized guidelines contributes to global practice variability, with some surgeons favouring partial weight bearing despite evidence supporting early full weight bearing. Hence, future research should focus on well-designed, large-scale randomized controlled trials with standardized protocols and long-term follow-up to establish definitive guidelines and address patient-specific factors. Standardized weight-bearing protocols for distinct clinical sub-groups are essential to optimize rehabilitation, reduce complications, and improve quality of life with neck of femur fractures.
